# Assessing Acceptance and Intention to Use of Home Telemonitoring Among Patients with Liver Cirrhosis: Development and Initial Psychometric Validation of a TAM-Based Questionnaire

**DOI:** 10.3390/diagnostics16162635

**Published:** 2026-08-19

**Authors:** Ruxandra-Cristina Marin, Horia Păunescu, Călin Muntean, Daniel Vasile Balaban, Laura Ioana Coman, Nicoleta Radu, Mariana Jinga, Oana Andreia Coman

**Affiliations:** 1 Discipline of Pharmacology, Clinical Pharmacology and Pharmacotherapy, “Carol Davila” University of Medicine and Pharmacy, 020021 Bucharest, Romania; ruxandra.marin@umfcd.ro (R.-C.M.); horia.paunescu@umfcd.ro (H.P.); oana.coman@umfcd.ro (O.A.C.); 2 Department III, Functional Science, Discipline of Medical Informatics and Biostatistics, “Victor Babes” University of Medicine and Pharmacy, 300041 Timisoara, Romania; 3 Faculty of Medicine, Internal Medicine and Gastroenterology Discipline, “Carol Davila” University of Medicine and Pharmacy, 020021 Bucharest, Romania; mariana.jinga@umfcd.ro; 4 Gastroenterology Department, “Dr. Carol Davila” Central Military University Emergency Hospital, 010825 Bucharest, Romania; 5 Medical Center for Diagnosis, Outpatient Treatment and Preventive Medicine, 011794 Bucharest, Romania; laura.coman21@yahoo.com; 6 Faculty of Biotechnology, University of Agronomic Sciences and Veterinary Medicine of Bucharest, 011464 Bucharest, Romania; nicoleta.radu@biotehnologii.usamv.ro; 7 National Department of Biotechnology, National Research and Development Institute for Chemistry and Petrochemistry–ICECHIM Bucharest, 202 Splaiul Independenței, 060021 Bucharest, Romania

**Keywords:** telemedicine, telemonitoring, liver cirrhosis, Technology Acceptance Model, psychometric validation, questionnaire, digital health, Romania

## Abstract

**Background/Objectives:** Liver cirrhosis (LC) is a major cause of morbidity and mortality requiring continuous monitoring and early recognition of clinical deterioration. Home telemonitoring is increasingly considered a supportive strategy for outpatient hepatology care; however, patient acceptance remains insufficiently characterized in Eastern Europe, and no validated instrument currently exists for this population. This study aimed to develop and provide the initial psychometric validation of a Technology Acceptance Model (TAM)-based questionnaire assessing acceptance of home telemonitoring among patients with LC. **Methods:** In this prospective cross-sectional study, 130 adult patients with LC (Child–Pugh A/B/C) were enrolled. A 25-item questionnaire, including 13 Likert-scale items mapped to Perceived Usefulness (PU), Perceived Ease of Use (PEU), and Intention to Use (ITU), was administered under supervision. Psychometric evaluation included internal consistency, exploratory and confirmatory factor analyses, structural equation modelling (SEM), convergent and discriminant validity, and known-groups validity. **Results:** Internal consistency was excellent (Cronbach’s α = 0.934; McDonald’s ω = 0.950). EFA identified a 2-factor structure explaining 77.9% of the variance, whereas CFA supported the theoretically driven 3-factor TAM, which showed improved incremental fit (CFI = 0.927; TLI = 0.908) and was retained on theoretical grounds. SEM confirmed the TAM pathway, with PU strongly predicting ITU (β = 0.823, *p* < 0.001), whereas PEU significantly influenced PU (β = 0.343, *p* < 0.001) but not ITU directly. The model explained 69.5% of the variance in ITU. Known-groups validity was supported across digital access, IT comfort, education level, and disease severity. Patients with advanced cirrhosis and lower digital confidence showed lower acceptance scores. **Conclusions:** The proposed 13-item Likert scale demonstrated good initial psychometric performance and may represent a useful tool for assessing telemonitoring acceptance and readiness for digital monitoring among patients with LC. Pending further multicentre validation and assessment of predictive validity, the instrument may support identification of patients requiring additional support for successful telemonitoring adoption.

## 1. Introduction

Chronic liver disease constitutes a major and escalating global health challenge, responsible for approximately 2 million deaths annually, representing nearly 4% of all-cause mortality worldwide and ranking among the leading causes of death globally. Of these deaths, around 1.3 million are attributed to liver cirrhosis (LC) and approximately 800,000 to hepatocellular carcinoma, highlighting the disproportionate contribution of advanced liver disease stages to overall mortality. In addition to mortality, cirrhosis contributes substantially to disability, ranking among the top causes of disability-adjusted life-years (DALYs) in individuals aged 25–49 years, thereby exerting a significant socioeconomic impact. Furthermore, global trends indicate a steady increase in cirrhosis-related deaths of nearly 50% since 1990, with the highest burden observed in Eastern Europe, reflecting persistent exposure to modifiable risk factors and disparities in access to healthcare services [1,2].

In Europe, alcohol-related liver disease remains a major cause of cirrhosis, with Romania reporting among the highest levels of alcohol consumption in the WHO European Region [3,4]. Meanwhile, viral etiologies are declining following hepatitis B vaccination and effective hepatitis C antiviral therapy [5,6], whereas metabolic dysfunction-associated steatotic liver disease (MASLD) is increasingly contributing to the chronic liver disease burden [7]. In Romania, LC remains the leading cause of mortality among digestive diseases, with higher mortality rates in rural populations, highlighting inequalities in access to preventive and specialized healthcare services [8].

A substantial burden of liver disease remains undiagnosed, particularly in high-risk groups; Romanian population-based data identified a high prevalence of previously unrecognized advanced fibrosis and cirrhosis among asymptomatic individuals, supporting the role of non-invasive screening strategies in vulnerable populations [9]. Non-invasive methods, including transient elastography, can detect significant fibrosis in individuals without known liver disease, enabling earlier risk stratification and intervention [10]. Collectively, these data suggest that the true burden of liver disease may be substantially underestimated and underscore the need for improved strategies for early detection and longitudinal monitoring.

Given the need for longitudinal surveillance in cirrhosis, telemonitoring may complement conventional outpatient care by facilitating repeated assessment of symptoms and clinical parameters and enabling earlier recognition of clinically relevant deterioration [11,12].

Cirrhosis is a chronic, progressive condition characterized by significant morbidity, frequent complications, and high healthcare utilization. The transition from compensated to decompensated cirrhosis, marked by the development of complications such as ascites, hepatic encephalopathy, variceal bleeding, and spontaneous bacterial peritonitis, is associated with a dramatic reduction in survival and increased hospitalization rates. Recurrent hospital admissions and long-term management requirements contribute to a substantial economic burden on healthcare systems, making cirrhosis a paradigmatic example of a high-cost chronic disease requiring continuous care [2].

Given the complexity of cirrhosis management, contemporary care models emphasize continuous monitoring, early detection of decompensation, patient education, and adherence to long-term therapy. These characteristics make cirrhosis particularly suitable for digital health solutions, especially home-based telemonitoring systems supporting proactive and personalized care. Telemedicine has rapidly expanded in hepatology, particularly after the COVID-19 pandemic, highlighting the feasibility of maintaining continuity of care outside traditional clinical settings. In Romania, telemedicine-based management of chronic hepatitis C during lockdown also proved feasible and effective in maintaining treatment continuity [12,13,14].

Home telemonitoring can support longitudinal assessment of symptoms and selected clinical parameters, facilitate communication between patients and healthcare providers, and potentially enable earlier identification of clinical deterioration between scheduled visits [15].

Emerging digital tools have been applied to support specific aspects of cirrhosis management, including nutritional monitoring and personalized dietary interventions, further highlighting the potential of integrated digital health solutions [16]. Combined with patient education, telemonitoring-enabled self-management may improve LC management and reduce disease burden. Several studies, particularly during the COVID-19 pandemic, have demonstrated the feasibility, utility, and high patient satisfaction associated with telemedicine interventions in LC across different healthcare settings [17,18,19]. From a diagnostic perspective, telemonitoring systems may enhance longitudinal clinical assessment by enabling repeated patient-reported symptom collection and real-time communication of clinically relevant changes between scheduled visits, supporting continuity of care in a disease characterized by fluctuating clinical trajectories and recurrent exacerbations [20].

Despite these advantages, the successful implementation of telemonitoring interventions depends largely on patient acceptance and willingness to use these technologies. Adoption of digital health tools is influenced by factors such as perceived benefits, usability, prior experience with technology, socio-demographic characteristics, and social beliefs. Understanding these determinants is essential for the effective design and implementation of patient-centered telehealth interventions, highlighting the importance of behavioral models in predicting technology adoption [21,22]. In healthcare settings, Technology Acceptance Model (TAM)-based instruments may provide clinically useful information regarding the feasibility of implementing digital diagnostic and monitoring strategies in vulnerable patient populations. This is particularly relevant in chronic liver disease, where reduced adherence, limited digital literacy, cognitive impairment, and socioeconomic disparities may influence the effectiveness of remote monitoring programs, continuity of care, and long-term prognosis [23,24].

TAM, originally proposed in 1989 as a refinement of the Theory of Reasoned Action (TRA), is a widely recognized framework for predicting users’ acceptance of information technology. Within this model, behavioral intention to use a system, referred to as Intention to Use (ITU), is primarily determined by two key constructs: Perceived Usefulness (PU), defined as the extent to which an individual believes that using the system will improve outcomes or performance, and Perceived Ease of Use (PEU), defined as the degree to which the system is perceived as effortless to use. These constructs are considered the main predictors of ITU and have been consistently validated across diverse healthcare settings, establishing TAM as one of the most widely used models for understanding technology adoption [25].

Recent systematic reviews confirm that PU, PEU, attitude toward use, compatibility, self-efficacy, anxiety, and facilitating conditions are among the most relevant determinants of telemedicine acceptance, reinforcing the applicability of TAM constructs in digital health contexts [21,22].

According to TAM, ITU is mainly influenced by PU and PEU. However, real-world technology adoption may also be shaped by factors such as digital skills, previous experience with technology, anxiety, social influences, organizational support, and satisfaction with healthcare services, suggesting that extended TAM frameworks may better capture the complexity of healthcare technology adoption [26]. In the present study, TAM was preferred over extended frameworks such as TAM2 and the Unified Theory of Acceptance and Use of Technology (UTAUT) because its parsimonious structure minimizes respondent burden and is well suited to a predominantly older, clinically vulnerable population [27].

Applying TAM to patients with LC requires specific psychometric adaptation, as this population may include older adults, patients with multimorbidity, recurrent hospitalizations, cognitive impairment related to hepatic encephalopathy, and variable digital literacy. Therefore, questionnaire items should reflect both the clinical context of LC and the specific target technology, namely home telemonitoring. Contemporary recommendations for measurement-instrument development emphasize conceptual definition, item generation, expert review, pilot testing, and assessment of validity and reliability to ensure methodological rigor and reproducibility of findings [28,29,30].

To date, evidence regarding telemonitoring acceptance among patients with LC remains limited, and no TAM-based instrument appears to have been specifically validated for this population in the Romanian healthcare context. This gap is clinically relevant given the high burden of LC, the need for long-term outpatient monitoring, and the potential role of telemonitoring in supporting longitudinal care [31,32].

Accordingly, this study aims to support the assessment of patients’ acceptance of home telemonitoring systems in LC by developing and providing the initial psychometric validation of a TAM-based questionnaire. The instrument is intended to identify factors influencing patients’ willingness to use telemonitoring, such as perceived usefulness, ease of use, and individual characteristics, and may help healthcare professionals recognize potential barriers, select suitable patients, and adapt digital interventions to patient needs. Secondary objectives include evaluating internal consistency, exploring factor structure through exploratory factor analysis (EFA), testing competing measurement models using confirmatory factor analysis (CFA), examining relationships between TAM constructs through structural equation modeling (SEM), and assessing known-groups validity across clinically relevant subgroups, including device ownership, IT comfort, education level, and Child–Pugh class.

## 2. Materials and Methods

### 2.1. Study Design

A prospective, observational, cross-sectional study was conducted to develop and perform initial psychometric validation of a questionnaire assessing acceptance of, and intention to use, home telemonitoring among patients with LC.

The study was carried out at a tertiary-care hepatology center in Romania between September 2022 and June 2023. The study protocol was approved by the Ethics Committee of the Central Military Emergency University Hospital “Dr. Carol Davila” (approval no. 487/28 January 2022). All participants provided written informed consent before enrolment, and the study was conducted in accordance with the Declaration of Helsinki.

### 2.2. Participants and Data Collection

A total of 158 patients with a confirmed diagnosis of LC were screened for eligibility. Of these, 22 were excluded (severe hepatic encephalopathy, acute medical instability, or inability to complete the questionnaire), and 6 declined participation. The final sample comprised 130 consecutive adult patients recruited from the outpatient clinic and inpatient ward.

Inclusion criteria were confirmed diagnosis of LC based on clinical, biochemical, imaging, and/or histological criteria; age ≥ 18 years; ability to understand and complete the questionnaire; and provision of written informed consent. Exclusion criteria included: age under 18 years, severe cognitive impairment or hepatic encephalopathy precluding questionnaire completion, acute medical instability, and refusal to participate. Disease severity was classified according to the Child–Pugh score (A, B, or C) [33]. The MELD 3.0 score was not systematically recorded at enrolment and could therefore not be analysed as a continuous severity measure in the present validation study.

The questionnaire was administered in a supervised setting and was self-completed whenever possible. Assistance was provided by trained interviewers for patients with limited literacy or visual impairment, without influencing responses. Administration followed a standardized written protocol, and all interviewers received joint training to read items verbatim and to provide only neutral, non-directive clarifications, ensuring consistency of assistance across interviewers. All questionnaires were checked for completeness at the time of administration (i.e., the attitudinal scale subjected to psychometric validation). No missing data were observed for the 13 Likert-scale items; therefore, no imputation was required. Beyond complete-case analysis, no imputation procedures were prespecified, as the supervised administration was designed to prevent missing responses. The sample size exceeded the recommended minimum of five participants per item for factor analysis. Because this criterion is primarily intended for exploratory analyses, the sample was considered adequate for EFA, whereas the CFA and SEM results should be regarded as preliminary, since these models generally require larger samples for stable parameter estimates.

### 2.3. Questionnaire Development and Content Validation

The questionnaire was developed following a structured multi-stage process aligned with COSMIN guidelines (Table 1) [34,35].

The process included: (1) definition of the conceptual framework, (2) item generation, (3) expert-based content validation (multidisciplinary panel, n = 6), (4) questionnaire structuring, (5) pilot testing for face validity (n = 15), (6) finalization, and (7) psychometric evaluation.

The instrument was grounded in the TAM, focusing on PU, PEU, and ITU [36].

The final questionnaire comprised 25 items organized into four domains: socio-demographic characteristics (Q1–Q4), digital access and literacy (Q5–Q8), prior telemedicine exposure (Q9–Q12), and a core attitudinal scale (Q13–Q25). While the full questionnaire contained 25 items, psychometric validation was restricted to 13 Likert-type attitudinal items because only these items were designed as reflective indicators of latent TAM-related constructs. The remaining 12 items captured socio-demographic, clinical, digital-access, and prior-experience variables and were therefore used for descriptive and known-groups validity analyses rather than internal consistency or factor-analytic validation.

The core scale included 13 Likert-type items rated on a five-point scale (1 = strongly disagree; 5 = strongly agree): PU (Q13–Q16), PEU (Q17–Q20; Q19 and Q20 reverse-coded), IT self-efficacy (Q21), ITU (Q22), social support (Q23), trust/privacy (Q24), and preference for home monitoring (Q25).

The full questionnaire (English translation) is provided in Supplementary File S1.

### 2.4. Statistical Analysis

Descriptive statistics were computed for all items, including means, standard deviations, skewness, kurtosis, and floor/ceiling effects. A floor or ceiling effect was defined a priori as >15% of responses at the lowest or highest category [37]. Normality was assessed using the Shapiro–Wilk test. Skewness and kurtosis were evaluated against the thresholds |skewness| ≤ 2 and |kurtosis| ≤ 7 when judging the appropriateness of maximum-likelihood estimation.

Internal consistency was evaluated using Cronbach’s α and McDonald’s ω, along with corrected item–total correlations. Values ≥ 0.70 were considered acceptable [38,39].

Sampling adequacy was assessed using the Kaiser–Meyer–Olkin (KMO) index and Bartlett’s test of sphericity. Exploratory factor analysis (EFA) was performed using principal-axis factoring with promax rotation. The number of factors was determined based on Horn’s parallel analysis and eigenvalues > 1 [40,41].

Confirmatory factor analysis (CFA) was conducted to compare a two-factor model derived from EFA with a theoretically driven three-factor TAM (PU, PEU, ITU). Model fit was evaluated using the comparative fit index (CFI), Tucker–Lewis index (TLI), root mean square error of approximation (RMSEA), standardized root mean square residual (SRMR), and χ^2^ statistic [42,43]. Parameters were estimated using maximum likelihood with robust standard errors, an approach with acceptable performance for five-category Likert-type indicators under moderate non-normality [44]. Given the ordinal nature of the items, estimation using weighted least squares with mean and variance adjustment (WLSMV) is planned as a sensitivity analysis in the subsequent, larger-sample validation stage [45].

Structural equation modelling (SEM) was used to test the TAM pathway (PEU → PU; PU → ITU; PEU → ITU), with standardized path coefficients and explained variance (R^2^) reported.

Convergent validity was assessed using average variance extracted (AVE ≥ 0.50) and composite reliability (CR ≥ 0.70), while discriminant validity was evaluated using the Fornell–Larcker criterion. Known-groups validity was examined using Mann–Whitney U tests and Kruskal–Wallis H tests with Bonferroni-adjusted post hoc comparisons; when the omnibus test was significant, pairwise group contrasts (e.g., Child–Pugh A vs. B, A vs. C, and B vs. C) were examined using Mann–Whitney U tests with Bonferroni-corrected *p*-values. Effect sizes were reported as rank-biserial r for binary comparisons and η^2^ for multi-group comparisons. Associations with age were assessed using Spearman’s ρ.

Statistical analyses were performed using R version 4.3.2 within RStudio, including packages for psychometric analysis and structural equation modelling (e.g., lavaan). Statistical significance was set at α = 0.05 (two-sided).

## 3. Results

### 3.1. Sample Characteristics

Of 158 patients screened, 22 were excluded (11 with severe encephalopathy, 5 with acute medical instability, 6 with inability to complete the questionnaire) and 6 declined participation, resulting in a final sample of 130 patients. The mean age was 62.2 ± 8.6 years (range 39–79), and 60.8% were male. Alcohol-related cirrhosis was the most common etiology (49.2%). Detailed demographic, clinical, and digital-access characteristics are presented in Table 2.

### 3.2. Descriptive Statistics and Distributional Properties of Likert Items

Descriptive statistics for the 13 Likert-scale items are presented in Table 3. Items assessing perceived usefulness (Q13–Q16) showed high mean scores (4.30–4.38) with substantial ceiling effects (53–61%), indicating a strong perceived benefit of telemonitoring. Items related to perceived ease of use (Q17–Q20) and IT self-efficacy (Q21) demonstrated greater variability (means 3.05–3.56).

Applying the predefined 15% threshold, ceiling effects were observed for all 13 items, being most pronounced for Q13–Q16 and Q22–Q25 (44.6–60.8%) and more modest for Q17–Q21 (16.9–40.8%); floor effects exceeding 15% were additionally observed for Q17, Q18, Q20, and Q21. All items showed significant deviations from normality (Shapiro–Wilk *p* < 0.001); however, skewness and kurtosis remained within acceptable limits for maximum-likelihood estimation (|skewness| ≤ 2; |kurtosis| ≤ 7).

### 3.3. Internal Consistency, Convergent and Discriminant Validity

The overall scale demonstrated excellent internal consistency (Cronbach’s α = 0.934; McDonald’s ω = 0.950). Subscale reliability was also high (PU: α = 0.985; PEU: α = 0.932; ITU: α = 0.864). Corrected item–total correlations ranged from 0.558 to 0.815, and removal of any item did not meaningfully improve internal consistency, supporting retention of all items. The very high reliability of the PU subscale suggests potential item redundancy, which will be considered in subsequent refinement stages.

Convergent validity was supported for all constructs, with average variance extracted (AVE) ranging from 0.720 to 0.956 and composite reliability (CR) exceeding 0.90. Discriminant validity was confirmed using the Fornell–Larcker criterion: the square root of AVE exceeded inter-construct correlations for all construct pairs. Combined reliability and validity indices are presented in Table 4.

### 3.4. Exploratory Factor Analysis

Sampling adequacy was high, as indicated by the Kaiser–Meyer–Olkin measure (KMO = 0.870), and Bartlett’s test of sphericity was significant (χ^2^(78) = 2163.57, *p* < 0.001), confirming the suitability of the data for factor analysis.

Factor retention was guided by parallel analysis, which supported a two-factor solution. The extracted factors accounted for 77.9% of the total variance (Factor 1: 46.0%; Factor 2: 32.0%).

Exploratory factor analysis was conducted using principal-axis factoring with promax (oblique) rotation. The resulting solution demonstrated a clear and interpretable two-factor structure (Table 5). Factor 1, labelled Perceived Value and Acceptance, comprised items related to perceived usefulness, intention to use, social support, trust/privacy, and preference for home monitoring (Q13–Q16, Q22–Q25). Factor 2, labelled Digital Competence, included items reflecting perceived ease of use and IT self-efficacy (Q17–Q21).

Factor loadings were strong and well-defined, with all primary loadings exceeding the prespecified threshold of 0.40. Communalities were acceptable across items, indicating that a substantial proportion of item variance was explained by the extracted factors. The correlation between factors was moderate (φ = 0.502), supporting the appropriateness of oblique rotation.

Minor cross-loadings were observed for Q19 and Q25; however, both items were retained within their theoretically assigned factors, as their primary loadings were higher and conceptually consistent.

### 3.5. Confirmatory Factor Analysis

Two competing measurement models were evaluated: a two-factor model derived from the exploratory factor analysis (EFA) and the hypothesized three-factor Technology Acceptance Model (TAM) structure, comprising perceived usefulness (PU), perceived ease of use (PEU), and intention to use (ITU).

The three-factor TAM demonstrated improved fit relative to the two-factor solution (ΔCFI = 0.032; ΔTLI = 0.035) (Table 6).

Although tests of normality were significant (Shapiro–Wilk *p* < 0.001), skewness and kurtosis values were within acceptable thresholds for maximum-likelihood estimation (|skew| ≤ 2; |kurtosis| ≤ 7), supporting the use of ML-based CFA.

Incremental fit indices for the TAM met recommended thresholds (CFI = 0.927; TLI = 0.908), and SRMR (0.063) indicated acceptable residual fit. RMSEA was elevated (0.141; 90% CI 0.121–0.162); however, given the moderate sample size and the presence of skewed Likert-type indicators with ceiling effects, this index may be inflated and should be interpreted alongside incremental fit indices. Simulation studies show that RMSEA can be markedly inflated when skewed, ordinal indicators are analysed, particularly with maximum-likelihood-based estimation in moderate samples [44,45].

Overall, the results provide acceptable support for the hypothesized three-factor TAM structure.

**Figure 1:**
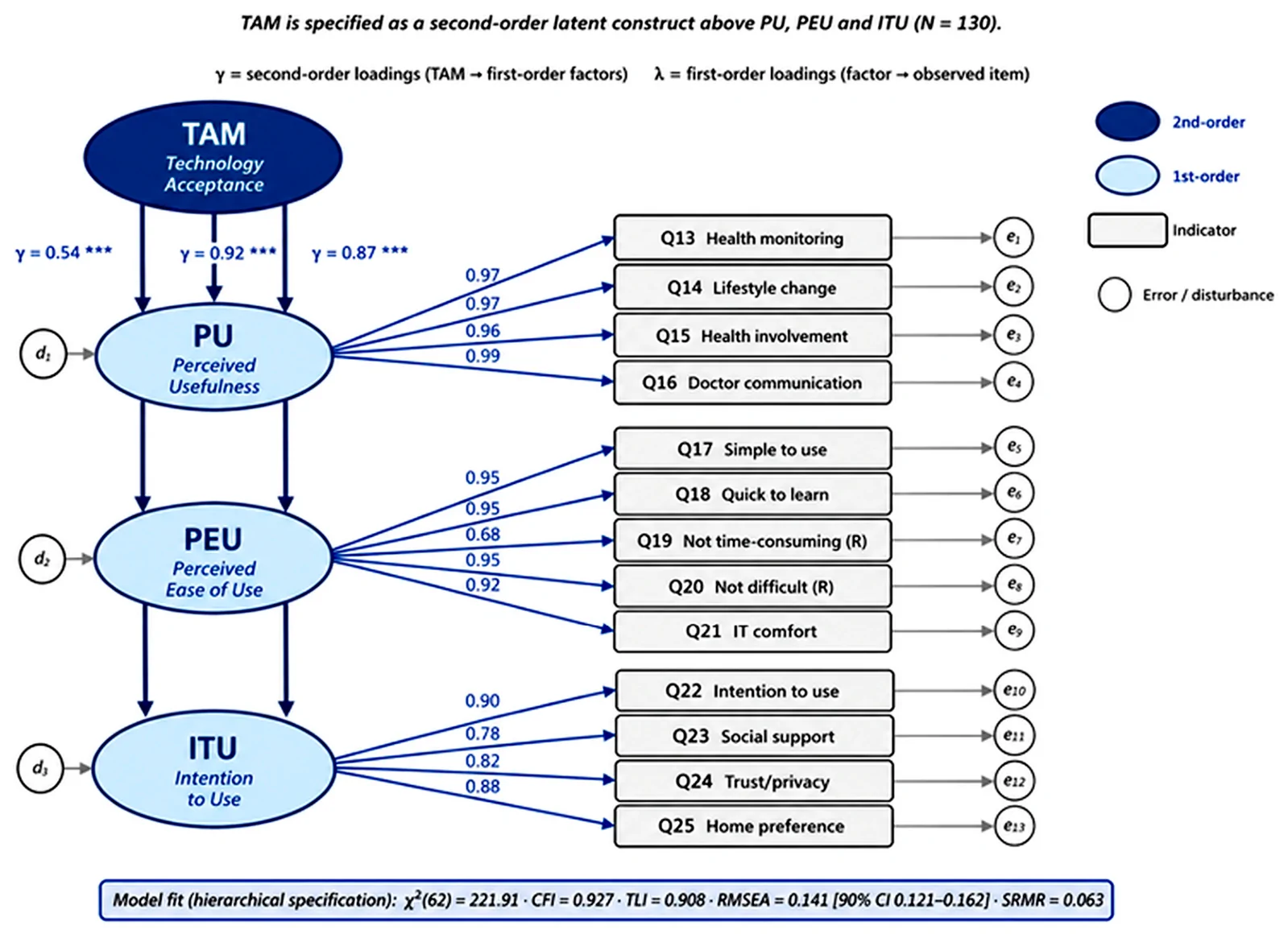
Confirmatory factor analysis of the hierarchical TAM. Standardized factor loadings for the three-factor Technology Acceptance Model (TAM). The model includes three latent constructs: perceived usefulness (PU), perceived ease of use (PEU), and intention to use (ITU). Latent variables are represented by ellipses and observed indicators by rectangles; measurement errors are shown as small circles. All factor loadings are statistically significant (*p* < 0.001). Model fit: χ^2^(62) = 221.91; CFI = 0.927; TLI = 0.908; RMSEA = 0.141 (90% CI 0.121–0.162); SRMR = 0.063. With three first-order factors, the hierarchical (second-order) specification is a just-identified reparameterization of the correlated three-factor model; its fit is mathematically identical to that of the first-order model, and no incremental superiority is implied; ***, *p* < 0.001.

Standardized factor loadings for the TAM are presented in Table 7 and illustrated in Figure 1. All loadings were statistically significant (*p* < 0.001), with values ranging from 0.684 to 0.988.

### 3.6. Structural Equation Modelling (TAM Pathway)

**Figure 2:**
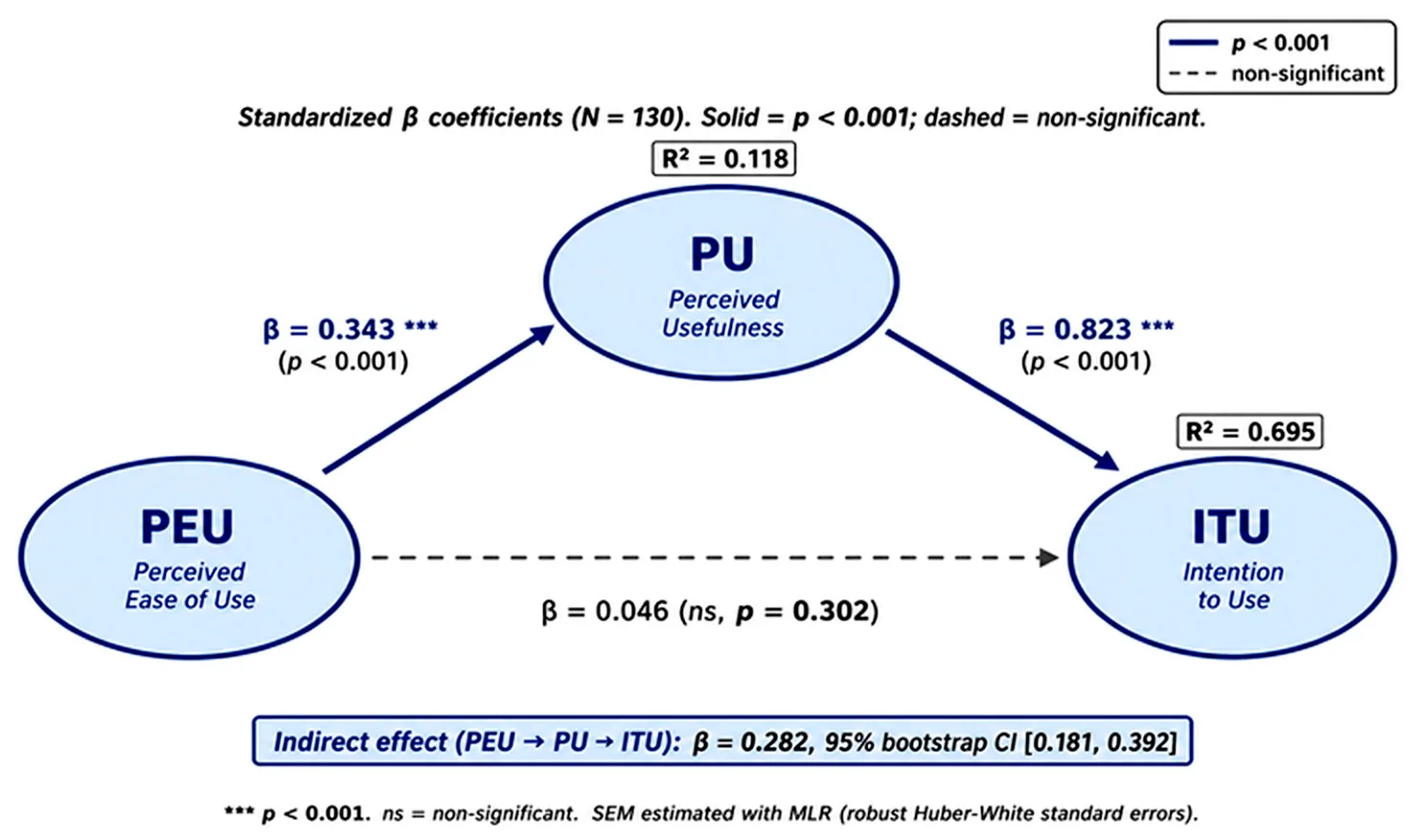
Structural equation model of the TAM pathway. Standardized path coefficients (β) for the relationships between perceived ease of use (PEU), perceived usefulness (PU), and intention to use (ITU). Solid arrows indicate statistically significant paths (*p* < 0.001), and dashed arrows indicate non-significant paths. R^2^ values represent explained variance for endogenous constructs. The indirect effect (PEU → PU → ITU) was significant (β = 0.282; 95% CI [0.181, 0.392]), consistent with full mediation. Model estimated using maximum likelihood with robust standard errors.

Structural equation modelling results are presented in Table 8 and Figure 2. Perceived usefulness was the strongest predictor of intention to use (β = 0.823, *p* < 0.001), while perceived ease of use significantly predicted perceived usefulness (β = 0.343, *p* < 0.001).

The direct effect of perceived ease of use on intention to use was not significant (β = 0.046, *p* = 0.302), indicating that its influence is primarily indirect. Mediation analysis confirmed a significant indirect effect (β = 0.282; 95% CI [0.181, 0.392]), consistent with full mediation.

The model explained 11.8% of the variance in perceived usefulness and 69.5% of the variance in intention to use, indicating strong explanatory power.

### 3.7. Known-Groups Validity

Known-groups validity analyses, combining Mann–Whitney U tests for binary group comparisons and Kruskal–Wallis H tests for multi-group comparisons, are summarized in Table 9. Patients owning a digital device, those reporting IT comfort, and those with prior telemedicine use scored significantly higher across most subscales, with the largest effect for PEU among IT-comfort comparisons (r = 0.54). No significant differences were observed by gender. Child–Pugh class was significantly associated with PU (H = 11.58, *p* = 0.003), PEU (H = 8.61, *p* = 0.014), and overall acceptance (H = 12.25, *p* = 0.002), but not with ITU; Bonferroni-adjusted post hoc comparisons indicated significantly lower PU and overall acceptance in class C compared with classes A and B (*p* < 0.05). Education level was associated with PEU and overall acceptance. Age showed modest negative correlations with PEU (ρ = −0.215; *p* = 0.014) and overall acceptance (ρ = −0.195; *p* = 0.026), indicating lower acceptance among older patients.

## 4. Discussion

This study describes the development and initial psychometric validation of a TAM-based questionnaire assessing acceptance of, and intention to use, home telemonitoring among patients with LC. The findings support the preliminary reliability and construct validity of the instrument and confirm that the Technology Acceptance Model provides an appropriate theoretical framework for evaluating telemonitoring acceptance in this population. This is consistent with prior healthcare technology acceptance literature, demonstrating that perceived usefulness and perceived ease of use are central determinants of behavioral intention [22]. To our knowledge, this is the first TAM-based instrument developed and subjected to initial psychometric evaluation specifically for assessing acceptance of home telemonitoring among patients with LC in an Eastern European healthcare setting. Unlike generic TAM-based instruments applied across broader telemedicine or digital-health contexts, the present questionnaire was developed specifically for the clinical and implementation context of cirrhosis, combining core TAM constructs with contextual information on digital access, prior telemedicine experience, and clinically relevant patient characteristics. This disease-specific contextualization may facilitate the identification of barriers to telemonitoring adoption that are particularly relevant to patients with LC.

The high mean scores and ceiling effects observed for perceived usefulness items suggest a generally favorable attitude toward telemonitoring. While this may indicate that patients recognize the potential clinical benefits of such systems, it should be interpreted with caution, as ceiling effects may also reflect limited variability or response bias rather than true pre-existing acceptance. Similar patterns have been reported in digital health studies, where perceived usefulness tends to dominate early acceptance, particularly in populations with high disease burden [46]. Ceiling effects of this magnitude also raise the possibility of social desirability bias, whereby patients endorse the general principle that health monitoring is beneficial rather than appraising the specific technology under evaluation. The questionnaire demonstrated strong internal consistency across all domains. However, the very high reliability observed for the perceived usefulness subscale may indicate partial item redundancy. According to measurement guidelines, internal consistency should be interpreted alongside content validity and structural evidence rather than as an isolated indicator of quality [28]. Item removal did not improve reliability, supporting retention of all items at this stage. This pattern is consistent with early-stage validation studies, where high internal consistency often reflects conceptual coherence but may require refinement in later validation phases [29]. Accordingly, subsequent validation should examine whether reformulation or reduction of highly overlapping PU items, together with refinement of the response scale, can improve discrimination at the upper end of the construct without compromising content validity.

EFA identified two dimensions, “Perceived Value and Acceptance” and “Digital Competence”, which together explained a large proportion of variance. This structure is clinically meaningful, as telemonitoring adoption likely depends on both perceived benefit and the ability to use the technology. Similar determinants, including perceived usefulness, usability, and self-efficacy, have been consistently identified in systematic reviews of telemedicine adoption [47]. The observed clustering of usefulness, intention, trust, and social support into a single evaluative dimension may reflect the way patients cognitively integrate these concepts in real-life decision-making, rather than treating them as strictly separate theoretical constructs. This phenomenon has been described in applied TAM research, where contextual and experiential factors influence how users perceive and group technology-related attributes [25,48].

CFA showed that the theoretically driven three-factor TAM performed better than the two-factor EFA-derived model, supporting the distinction between perceived usefulness, perceived ease of use, and intention to use. Although CFI and TLI suggested acceptable incremental fit, and SRMR was within an acceptable range, RMSEA was elevated. Therefore, the model should be interpreted as promising but not definitive. This cautious interpretation is appropriate because model fit indices may provide divergent information, and current CFA/SEM reporting recommendations emphasize evaluating several fit indices together rather than relying on a single statistic [42]. In addition, the presence of skewed Likert-type responses and ceiling effects may have contributed to the inflation of RMSEA, a limitation that has been acknowledged in recent SEM methodological literature, particularly in moderate sample sizes [43]. Consistent with simulation evidence on RMSEA inflation with ordinal, skewed indicators [44,45], a WLSMV-based sensitivity analysis will be undertaken in the planned larger-sample, independent validation.

Convergent validity was supported by high average variance extracted and composite reliability values, while discriminant validity was supported using the Fornell–Larcker criterion. These findings indicate that the three TAM constructs were internally coherent and sufficiently distinct in this sample. Nevertheless, because no external comparator instrument was administered, the present study supports internal construct validity but cannot establish external convergent validity. This distinction is important in line with COSMIN guidance for reporting measurement properties of patient-reported outcome measures [28]. Taken together, these results suggest that the instrument captures both the evaluative and functional dimensions of technology acceptance in patients with LC. The potential clinical utility of the instrument for guiding digital surveillance strategies remains to be demonstrated in prospective implementation studies [32,49].

Structural equation modelling confirmed the expected TAM pathway. Perceived usefulness was the strongest predictor of intention to use, while perceived ease of use influenced intention indirectly through perceived usefulness. This suggests that usability contributes to adoption primarily by enhancing the perceived clinical value of telemonitoring. This pattern aligns with healthcare technology acceptance research, where perceived usefulness consistently emerges as the dominant determinant of behavioral intention [22,50].

From an implementation perspective, the present findings suggest that perceived usefulness and ease of use may be relevant when assessing patients’ readiness for home telemonitoring. However, as this study evaluated hypothetical acceptance rather than actual use of an implemented telemonitoring system, the clinical utility of the instrument cannot yet be established. Prospective implementation studies are required to determine whether questionnaire scores predict actual telemonitoring uptake, adherence, sustained engagement, or clinically relevant outcomes [51,52].

In addition, emerging digital interventions, including mobile health applications and remote monitoring platforms, have demonstrated potential benefits in improving patient engagement and facilitating self-management in cirrhosis, further supporting the relevance of assessing patient acceptance prior to implementation [53].

The known-groups analyses further support the clinical interpretability of the questionnaire. Patients with device ownership, greater IT comfort, and previous telemedicine experience reported higher acceptance scores, particularly for perceived ease of use. This is consistent with broader evidence showing that digital access, confidence, and previous experience are important facilitators of digital health adoption, while lack of skills and limited access remain important barriers [54]. The moderate-to-large effect sizes observed in this study further suggest that these factors are not only statistically significant but also clinically meaningful in influencing telemonitoring readiness.

Older age was associated with lower perceived ease of use and lower overall acceptance. This finding is clinically relevant because cirrhosis frequently affects older patients, who may also experience physical limitations, cognitive impairment, and reduced digital confidence. Recent evidence on telehealth use among older adults highlights digital literacy, access to devices, cognitive limitations, and the need for support as key factors influencing adoption [55]. Medical informatics literature emphasizes that aging populations require more personalized, accessible, and user-centered digital health technologies in order to support equitable adoption and sustained engagement [56]. The results reinforce the importance of age-adapted implementation strategies, particularly in populations with chronic and complex diseases.

Patients with Child–Pugh class C had lower perceived usefulness and overall acceptance than those with less advanced disease. This may reflect greater symptom burden, fatigue, cognitive impairment, reduced independence, or reliance on caregivers. Therefore, patients with advanced cirrhosis may require adapted implementation strategies and closer healthcare team support. This interpretation is consistent with recent cirrhosis-specific remote patient monitoring evidence showing that implementation in decompensated cirrhosis requires structured workflows and patient support [49]. This finding suggests that disease severity itself may act as a barrier to digital health adoption, highlighting the need for differentiated care pathways rather than a one-size-fits-all approach. These findings may also have prognostic relevance, as patients with advanced disease are often those who could benefit most from closer monitoring and earlier detection of complications, yet simultaneously represent a group at increased risk of digital exclusion due to frailty, cognitive impairment, or reduced autonomy. Consequently, telemonitoring implementation in advanced cirrhosis may require individualized support and caregiver-assisted approaches [57,58].

The presence of ceiling effects for perceived usefulness and trust/privacy items suggests that many patients had a favorable baseline attitude toward telemonitoring. While encouraging, this may reduce sensitivity to detect further improvement after an intervention. Future versions of the questionnaire may benefit from more discriminative response options or revised item wording. This is consistent with measurement-development guidance, which recommends evaluating interpretability issues such as floor and ceiling effects during instrument refinement [28].

Overall, the results suggest that the questionnaire may be useful both as a research tool and as a practical instrument to identify barriers before telemonitoring implementation. In clinical practice, it may help healthcare professionals identify patients who need additional explanation, technical support, or caregiver involvement. In research, it may support the evaluation of telemonitoring programs by measuring acceptance before and after implementation. Recent cirrhosis-specific digital health studies, including remote patient monitoring programs and mobile applications for patient education, support the growing interest in structured digital tools for cirrhosis care [53]. Identifying patients with lower readiness for telemonitoring could help clinicians deliver targeted educational or technical support before deployment of remote monitoring systems [12,59].

Several limitations should be acknowledged. The single-centre design and moderate sample size may limit generalizability and affect the stability of CFA estimates. Although the sample size was adequate for exploratory analysis, it may be considered relatively small for confirmatory modelling, which could influence fit indices and parameter estimates. Moreover, exploratory and confirmatory factor analyses were performed in the same sample, which may lead to overfitting and artificially optimistic model performance; independent-sample cross-validation is therefore required. Disease severity was characterized using Child–Pugh class only; modelling severity continuously with the MELD 3.0 score [60] may provide greater clinical granularity in future validation studies. In addition, the cross-sectional design does not allow assessment of changes over time or prediction of actual telemonitoring use, so causal inferences regarding the TAM pathway should be interpreted cautiously. Moreover, the study evaluated hypothetical acceptance of telemonitoring rather than real-world interaction with an implemented monitoring platform, which may differ from actual technology use behaviors. In addition, although telemonitoring acceptance may influence future implementation success, the present study did not evaluate objective clinical endpoints, diagnostic performance measures, or prognostic outcomes such as hospitalization, decompensation events, or mortality. Therefore, further longitudinal studies are necessary to determine whether higher acceptance scores are associated with improved adherence to remote monitoring systems and clinically meaningful outcomes [61].

Test–retest reliability was not evaluated, and no external comparator instrument was included, which limits conclusions regarding temporal stability and external validity; this reflects the exploratory nature of this initial validation phase and should be addressed in subsequent studies. Furthermore, the predominance of alcohol-related cirrhosis reflects the Romanian clinical context and may limit transferability to settings where viral or metabolic etiologies predominate, although this distribution is representative of regional epidemiology and supports the internal validity of the findings within this context. Patients with alcohol-related cirrhosis may nevertheless differ from other etiological groups in socioeconomic status, digital literacy, and engagement with healthcare services, which could influence TAM construct scores; etiology-stratified analyses were not feasible given subgroup sizes and should be undertaken in larger cohorts. Finally, the presence of ceiling effects may have reduced variability in key constructs, potentially attenuating associations and influencing model fit.

Future studies should validate the questionnaire in larger multicenter cohorts and in populations with different cirrhosis etiologies and healthcare settings. Further work should also assess test–retest reliability, responsiveness, external convergent validity, and predictive validity, particularly whether baseline acceptance scores are associated with actual telemonitoring uptake, adherence, and clinical outcomes.

## 5. Conclusions

The 13-item Likert scale embedded in the 25-item questionnaire showed promising initial psychometric performance for assessing telemonitoring acceptance among patients with LC, with strong internal consistency, a coherent TAM-consistent factor structure, and known-groups validity. PU was the main determinant of ITU, whereas PEU acted indirectly through PU. Pending multicentre validation and assessment of predictive validity, the instrument may help identify patients who require additional educational, technical, or caregiver support before home telemonitoring is implemented.

## Figures and Tables

**Table 1:** Questionnaire development and psychometric evaluation workflow.

Stage	Description
1. Conceptual framework	Based on TAM, targeting PU, PEU, and ITU in the context of telemonitoring among patients with liver cirrhosis.
2. Item generation	Initial item pool developed in Romanian based on a targeted literature review of telemedicine, telemonitoring, and existing TAM-based instruments; items adapted to the clinical context.
3. Content validation	A multidisciplinary expert panel (n = 6) with expertise in gastroenterology, psychology, and academic research evaluated item relevance, clarity, and conceptual alignment; items were revised based on consensus.
4. Questionnaire structuring	Items were organized into four domains: socio-demographics, digital access and literacy, prior telemedicine exposure, and core TAM-based attitudinal scale (Likert-type items).
5. Pilot testing	Face validity and comprehensibility were assessed in 15 patients with liver cirrhosis; feedback was used to refine wording and improve clarity.
6. Finalization	Minor linguistic revisions were applied; item structure and domain allocation remained unchanged.
7. Psychometric evaluation	Internal consistency (Cronbach’s α, McDonald’s ω), exploratory factor analysis (EFA), confirmatory factor analysis (CFA), structural equation modelling (SEM), convergent and discriminant validity, and known-groups validity were assessed.

TAM, Technology Acceptance Model; PU, perceived usefulness; PEU, perceived ease of use; ITU, intention to use; EFA, exploratory factor analysis; CFA, confirmatory factor analysis; SEM, structural equation modelling; n, number.

**Table 2:** Demographic, clinical and digital-access characteristics of the study sample (N = 130).

Characteristic	n (%)/Mean ± SD
Age (years), mean ± SD	62.2 ± 8.6
Gender—Male	79 (60.8)
Gender—Female	51 (39.2)
Education—Primary school	13 (10.0)
Education—Professional school	51 (39.2)
Education—High school	31 (23.8)
Education—University	35 (26.9)
Child–Pugh A	43 (33.1)
Child–Pugh B	40 (30.8)
Child–Pugh C	47 (36.2)
Etiology—Alcohol	64 (49.2)
Etiology—MASLD	20 (15.4)
Etiology—HBV	17 (13.1)
Etiology—HCV	14 (10.8)
Etiology—Other	15 (11.5)
Device ownership (Yes)	92 (70.8)
Wi-Fi at home (Yes)	104 (80.0)
IT comfort (Yes)	63 (48.5)
Prior telemedicine use (Yes)	25 (19.2)
Health apps (Yes)	16 (12.3)
Home monitoring devices (Yes)	40 (30.8)

Values are presented as mean ± standard deviation (SD) or number (%). SD, standard deviation; MASLD, metabolic dysfunction-associated steatotic liver disease; HBV, hepatitis B virus; HCV, hepatitis C virus; IT, information technology; Wi-Fi, wireless fidelity.

**Table 3:** Descriptive statistics of Likert-scale items (Q13–Q25).

Item	Content	Mean	SD	Skew	Kurt	Floor %	Ceiling %
Q13	Health monitoring	4.31	0.96	−1.56	1.96	1.5	53.8
Q14	Lifestyle change	4.30	0.95	−1.54	1.94	1.5	53.1
Q15	Health involvement	4.32	0.93	−1.60	2.25	1.5	53.8
Q16	Doctor communication	4.38	0.97	−1.69	2.20	1.5	60.8
Q17	Simple to use	3.05	1.50	−0.08	−1.53	20.8	21.5
Q18	Quick to learn	3.48	1.58	−0.45	−1.44	16.9	40.8
Q19	Not time-consuming (R)	3.56	1.06	−0.53	−0.67	2.3	16.9
Q20	Not difficult (R)	3.18	1.54	−0.32	−1.47	24.6	23.1
Q21	IT comfort	3.05	1.54	−0.19	−1.53	26.9	20.8
Q22	Intention to use	4.13	1.04	−1.27	0.93	2.3	44.6
Q23	Social support	4.25	1.00	−1.26	0.67	0.8	53.1
Q24	Trust/privacy	4.36	0.90	−1.48	1.72	0.8	56.9
Q25	Home preference	4.12	1.07	−1.01	−0.16	0.8	49.2

Reverse-coded items (Q19, Q20) are presented after recoding. Skew, skewness; Kurt, kurtosis; SD, standard deviation. Floor/Ceiling (%) indicate the proportion of lowest/highest responses.

**Table 4:** Reliability and validity indices for the TAM constructs (combined internal consistency, convergent and discriminant validity).

Construct	Items	Cronbach’s α	McDonald’s ω	AVE	CR	√AVE	PU	PEU	ITU
PU	4 (Q13–Q16)	0.985	0.987	0.956	0.989	0.978	—	0.502	0.801
PEU	5 (Q17–Q21)	0.932	0.940	0.788	0.948	0.888	0.502	—	0.474
ITU	4 (Q22–Q25)	0.864	0.881	0.720	0.911	0.849	0.801	0.474	—
Overall	13	0.934	0.950	—	—	—	—	—	—

PEU, perceived ease of use; PU, perceived usefulness; ITU, intention to use; AVE, average variance extracted; CR, composite reliability; √AVE, square root of AVE (Fornell–Larcker diagonal). Off-diagonal values (right block) are inter-construct correlations between PU, PEU and ITU. Discriminant validity is supported when √AVE exceeds inter-construct correlations.

**Table 5:** Exploratory factor analysis loading matrix (principal-axis factoring, promax rotation).

Item	Pattern F1	Pattern F2	Structure F1	Structure F2	h^2^ (Communality)
Q13	0.93 *	0.05	0.91 *	0.27	0.893
Q14	0.94 *	0.03	0.92 *	0.25	0.898
Q15	0.92 *	0.04	0.90 *	0.26	0.871
Q16	0.88 *	0.14	0.87 *	0.34	0.866
Q17	0.02	0.90 *	0.22	0.91 *	0.863
Q18	0.08	0.91 *	0.28	0.92 *	0.913
Q19 (R)	0.25	0.49 *	0.41	0.57 *	0.478
Q20 (R)	0.02	0.90 *	0.22	0.91 *	0.868
Q21	−0.05	0.93 *	0.17	0.91 *	0.861
Q22	0.83 *	0.11	0.84 *	0.28	0.774
Q23	0.78 *	−0.09	0.75 *	0.10	0.577
Q24	0.90 *	−0.15	0.86 *	0.03	0.737
Q25	0.58 *	0.23	0.63 *	0.38	0.531

Exploratory factor analysis was conducted using principal-axis factoring with promax (oblique) rotation. Pattern and structure coefficients are reported. Primary loadings (|λ| ≥ 0.40) are indicated with *. h^2^ (communality) represents the proportion of variance in each item explained by the extracted factors. The inter-factor correlation was φ(F1, F2) = 0.502. F1, Perceived Value and Acceptance (Q13–Q16, Q22–Q25); F2, Digital Competence (Q17–Q21). Total variance explained = 77.9% (F1 = 46.0%; F2 = 32.0%).

**Table 6:** Model fit indices for confirmatory factor analysis.

Model	χ^2^	df	CFI	TLI	RMSEA [90% CI]	SRMR	GFI
2-Factor (EFA-based)	293.51	64	0.895	0.873	0.167 [0.148–0.187]	0.082	0.871
3-Factor TAM (PU, PEU, ITU)	221.91	62	0.927	0.908	0.141 [0.121–0.162]	0.063	0.902

PEU, perceived ease of use; PU, perceived usefulness; ITU, intention to use; EFA, exploratory factor analysis; TAM, technology acceptance model; CFI, comparative fit index; TLI, Tucker–Lewis index; RMSEA, root mean square error of approximation; SRMR, standardized root mean square residual; GFI, goodness-of-fit index. GFI is reported for transparency only and was not used for model evaluation, as it is sensitive to sample size and is no longer recommended in contemporary SEM practice.

**Table 7:** Standardized factor loadings for the three-factor TAM.

Path	Std. λ	SE	z	*p*
PU → Q13	0.967	0.018	53.72	<0.001
PU → Q14	0.971	0.017	57.12	
PU → Q15	0.963	0.019	50.68	
PU → Q16	0.988	0.012	82.33	
PEU → Q17	0.948	0.021	45.14	
PEU → Q18	0.952	0.020	47.60	
PEU → Q19 (R)	0.684	0.066	10.36	
PEU → Q20 (R)	0.946	0.022	43.00	
PEU → Q21	0.920	0.026	35.38	
ITU → Q22	0.901	0.029	31.07	
ITU → Q23	0.782	0.047	16.64	
ITU → Q24	0.821	0.041	20.02	
ITU → Q25	0.877	0.033	26.58	

Standardized factor loadings (λ), standard errors (SE), z-values, and *p*-values are reported for the three-factor confirmatory factor analysis (CFA) model, comprising perceived usefulness (PU), perceived ease of use (PEU), and intention to use (ITU). All factor loadings were statistically significant (*p* < 0.001). (R) indicates reverse-coded items.

**Table 8:** Standardized structural equation model path coefficients (TAM pathway).

Path	β (Standardized)	SE	z-Value	*p*-Value
PEU → PU	0.343	0.056	6.12	<0.001
PU → ITU	0.823	0.069	11.94	
PEU → ITU (direct)	0.046	0.045	1.03	0.302

PEU, perceived ease of use; PU, perceived usefulness; ITU, intention to use; β, standardized path coefficient; SE, standard error.

**Table 9:** Known-groups validity (combined Mann–Whitney U and Kruskal–Wallis H tests).

Group Comparison (Expected Direction)	PU (*p*-Value, r/H)	PEU (*p*-Value, r/H)	ITU (*p*-Value, r/H)	Overall (*p*-Value, r/H)
Mann–Whitney U test (binary comparisons)				
Device Yes vs. No (Yes > No)	<0.001 ***, r = 0.44	<0.001 ***, r = 0.50	<0.001 ***, r = 0.30	<0.001 ***, r = 0.54
IT comfort Yes vs. No (Yes > No)	0.002 **, r = 0.26	<0.001 ***, r = 0.54	0.038 *, r = 0.17	<0.001 ***, r = 0.49
Prior telemedicine Yes vs. No (Yes > No)	0.002 **, r = 0.26	0.002 **, r = 0.27	0.112, ns	<0.001 ***, r = 0.30
Education High vs. Low (High > Low)	0.603, ns	0.006, r = 0.24	0.755, ns	0.016 *, r = 0.21
Kruskal–Wallis H test (multi-group comparisons)				
Gender M vs. F (no directional hypothesis)	ns, 0.205	Ns, 0.672	ns, 0.701	ns, 0.506
Child–Pugh (A, B, C)	0.003 **, H = 11.58	0.014 *, H = 8.61	ns, H = 1.30	0.002 **, H = 12.25
Education (4 levels)	0.014 *, H = 10.64	0.002 **, H = 14.88	ns, H = 7.78	<0.001 ***, H = 17.87,
Age groups	ns, H = 2.30	0.018 *, H = 8.00	ns, H = 1.68	0.027 *, H = 7.22

Values reported as *p*-value with rank-biserial r (MW) or H statistic with *p*-value (KW). PU, perceived usefulness; PEU, perceived ease of use; ITU, intention to use; ns, not significant; *, *p* < 0.05; **, *p* < 0.01; ***, *p* < 0.001.

## Data Availability

The data presented in this study are available on request from the first author. The data are not publicly available due to privacy and ethical restrictions.

## Supplementary Materials

The following supporting information can be downloaded at: https://www.mdpi.com/article/10.3390/diagnostics16162635/s1, File S1. Full Questionnaire (English translation).

## Abbreviations

The following abbreviations are used in this manuscript:

ALD	alcohol-related liver disease
AVE	average variance extracted
CFA	confirmatory factor analysis
CFI	comparative fit index
CR	composite reliability
DALY	disability-adjusted life-year
EFA	exploratory factor analysis
GFI	goodness-of-fit index
HBV	hepatitis B virus
HCV	hepatitis C virus
IT	information technology
ITU	intention to use
KMO	Kaiser–Meyer–Olkin
LC	liver cirrhosis
MASH	metabolic dysfunction-associated steatohepatitis
MASLD	metabolic dysfunction-associated steatotic liver disease
PEU	perceived ease of use
PU	perceived usefulness
RMSEA	root mean square error of approximation
SD	standard deviation
SEM	structural equation modelling
SRMR	standardized root mean square residual
TAM	Technology Acceptance Model
TLI	Tucker–Lewis index
TRA	Theory of Reasoned Action
WHO	World Health Organization
