# Supplementary material for: Assessing Acceptance and Intention to Use of Home Telemonitoring Among Patients with Liver Cirrhosis: Development and Initial Psychometric Validation of a TAM-Based Questionnaire
Source: Diagnostics (Basel). 2026 Aug 19;16(16):2635. doi: 10.3390/diagnostics16162635 (PMC13512894; doi:10.3390/diagnostics16162635)
Supplement: Supplementary file 1 [file diagnostics-16-02635-s001.zip › diagnostics-4353502-supplementary.pdf]

Supplementary File 1 - Full Questionnaire (English translation)

QUESTIONNAIRES ASSESSING THE ACCEPTANCE AND INTENTION TO USE INFORMATION TECHNOLOGY FOR REMOTE MONITORING, APPLIED TO PATIENTS WITH LIVER CIRRHOSIS

This questionnaire is confidential and anonymous and concerns only statistical processing aimed at assessing the opinion of patients with liver cirrhosis regarding home monitoring as an application of telemedicine.

The questionnaire will be completed after signing the attached informed consent.

Telemedicine represents the remote provision of medical services through technology (e.g. video conferencing, e-mail, phone call, text message, mobile application, etc.). This involves the transmission of medical data and information in the form of text, sound, image or other formats necessary for the purpose of preventing, diagnosing, treating a disease and/or following up with patients.

Telemonitoring is a branch of telemedicine, and involves remote monitoring of patients' health status. Data can be collected either automatically, through health monitoring devices (e.g. special medical sensors: blood pressure monitor, pulse oximeter, scale, thermometer, etc.), or based on the active collaboration of patients (e.g. by using an online tool to fill in daily weight or blood pressure values). Once processed and transmitted, the data can be used by physicians to optimize monitoring and treatment procedures.

Previous experience with telemedicine or home monitoring systems is not required to complete this questionnaire.

1) Age
□ 18-39
□ 40-49
□ 50-59
□ 60-69
□ ≥70

2) Gen
□ M
□ F

3) Education
□ primary school
□ high school
□ vocational school
□ university

4) Do you live alone?
□ Yes
□ No

5) Do you own a smartphone, tablet, personal computer or laptop?
□ Yes
□ No

6) Do you have a wi-fi connection at home?
□ Yes
□ No

7) If YES, how often do you use the above-mentioned devices?
□ daily
□ weekly (at least once a week)
□ monthly or less

8) Are you comfortable using information and communication technology? (e.g. internet, e-mail, video conferences, etc.)

□ Yes
□ No

9) **Have you ever benefited from telemedicine services? (e.g. teleconsultations, remote monitoring, etc.)**
□ Yes
□ No

10) **If YES, what type of telemedicine service did you use?**
□ video consultation
□ telephone consultation
□ E-mail consultation
□ health-related mobile app
□ remote monitoring app (home monitoring)
□ other (describe) ....

11) **Do you currently use any health-related mobile application?**
□ Yes
□ No

12) **Do you currently own/use any devices for monitoring health parameters? (e.g. smart watch, pulse oximeter, smart scale, etc.)**
□ Yes
□ No

13) **A telemonitoring system could help me better monitor my health.**
□ Totally Disagree □ Disagree □ Neutral □ Agree □ Totally Agree

14) **Using such a system could change my lifestyle for the better.**
□ Totally Disagree □ Disagree □ Neutral □ Agree □ Totally Agree

15) **If I were to use such a system, I would be more involved in improving my health.**
□ Totally Disagree □ Disagree □ Neutral □ Agree □ Totally Agree

16) **Monitoring my health at home would help me better communicate with my doctor.**
□ Totally Disagree □ Disagree □ Neutral □ Agree □ Totally Agree

17) **I believe that the use of such a system is simple and that it would not require permanent assistance.**
□ Totally Disagree □ Disagree □ Neutral □ Agree □ Totally Agree

18) **I could quickly learn how to use such a system if I received the necessary training and technical assistance.**
□ Totally Disagree □ Disagree □ Neutral □ Agree □ Totally Agree

19) **I think that the daily use of such a system would take up too much of my time**
□ Totally Disagree □ Disagree □ Neutral □ Agree □ Totally Agree

20) **I find that using such a system is difficult and that I would need permanent assistance throughout its use.**
□ Totally Disagree □ Disagree □ Neutral □ Agree □ Totally Agree

21) **I feel comfortable using IT devices (e.g. personal computer, laptop, tablet, smartphone, etc.)**
□ Totally Disagree □ Disagree □ Neutral □ Agree □ Totally Agree

22) **I intend to use such a system if it becomes available.**
□ Totally Disagree □ Disagree □ Neutral □ Agree □ Totally Agree

23) **I believe that my close ones would support me in using such a system.**
□ Totally Disagree □ Disagree □ Neutral □ Agree □ Totally Agree

24) **I am confident that the personal and medical data obtained from remote monitoring will be confidential and secure.**
□ Totally Disagree □ Disagree □ Neutral □ Agree □ Totally Agree

25) **I feel more comfortable having my health monitored at home than in the hospital.**
□ Totally Disagree □ Disagree □ Neutral □ Agree □ Totally Agree
